# Particle sizing in milk by combined differential dynamic microscopy and cryo-FIB-SEM tomography

**DOI:** 10.1039/d5sm01201e

**Published:** 2026-05-29

**Authors:** Joe J. Bradley, Fraser H. J. Laidlaw, Tom Pendry, Ngai Ying Denise Li, Alexander K. Boggon, Thomas Glen, Vincent A. Martinez, Jochen Arlt, Job H. J. Thijssen, Wilson C. K. Poon

**Affiliations:** a School of Physics & Astronomy, The University of Edinburgh Peter Guthrie Tait Road Edinburgh EH9 3FD UK joe@joe-bradley.co.uk W.Poon@ed.ac.uk; b Living Systems Institute, University of Exeter Exeter EX4 4QD UK; c The Rosalind Franklin Institute, Harwell Science & Innovation Campus Didcot OX11 0QS UK; d Dyneval Ltd, Roslin Innovation Center Edinburgh EH25 9RG UK

## Abstract

Milk is a suspension with a multimodal size distribution of fat droplets and protein micelles, which most sizing methods do not distinguish. We demonstrate the use of differential dynamic microscopy (DDM) and cryo-FIB-SEM tomography to size both fat globules and casein micelles in homogenised milk without the need for prior physical separation. The two techniques are complimentary: cryo FIB-SEM tomography can directly identify the 2 distinct constituents and reveal their overlapping size distributions. DDM reliably detects a bi-modal size distribution for whole milk samples, providing a fast high-throughput method to estimate volume-averaged mean sizes. Our results highlight that different sizing techniques seldom, if ever, yield the same answer. Instead, they can provide complementary information and further insights not obtainable from using each technique in isolation.

## Abstract 2

Size distributions for particles in milk as obtained by differential dynamic microscopy and cryo-focused ion beam-scanning electron microscopy.

## Graphical abstract

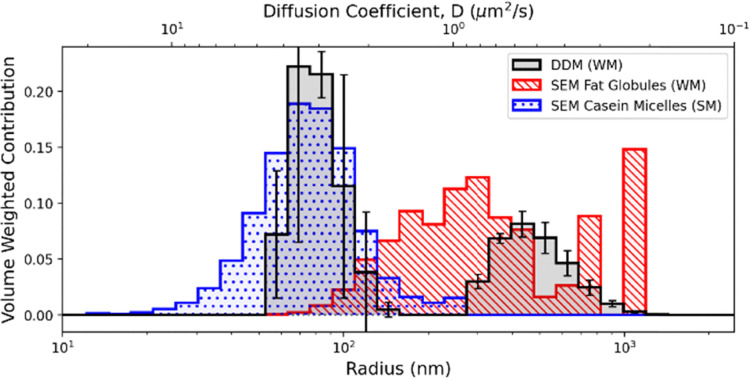

## 1 Introduction

In 1674, the inventor of the microscope Antonie van Leeuwenhoek^1^ discovered that ‘the sweet *Milk* of cow’ was ‘made up of small transparent globules… carried through a clear liquor’.^2^ We now know^3,4^ that these are fat droplets, which consist mostly of triglycerides enclosed by lipoprotein membranes. There is also a population of casein micelles of much smaller size. Casein^5^ make up ≈80% of the protein content in bovine milk, of which ≳75% are α- and β-casein micelles^6^ stabilised by κ-casein ‘hairs’.^7^ The fat and casein are dispersed in an aqueous solution of whey proteins^8^ and minerals, and show significant variability due to processing^9,10^ and biology.^11,12^

Accurately sizing the particles in milk is important in many applications, *e.g.*, homogenisation (fat droplet breakup)^13,14^ and cheesemaking (casein micelle aggregation),^15,16^ and in understanding the metabolic regulation of milk production.^17^ Light and X-ray scattering are among the commonest methods for such work,^18^ which often (but not always^19,20^) reports a bimodal size distribution for whole milk.^21–24^ Both modes are broad. The larger population peaks at††Sizes quoted in the Introduction are all diameters. ≲4 µm, but is rather sensitive to processing conditions.^9,10^ The smaller population is even more variable, typically peaking at ≲200 nm, although a value of ≈20 nm has been reported.^24^

As to the identity of the two populations detected by scattering, this conclusion is typical:^22^ ‘A bimodal characteristic distribution was found in raw milk: a 0.2 µm peak corresponding to casein micelles, and a second peak of 3.7 µm, corresponding to milk fat globules.’ This and similar conclusions go beyond the evidence, because the different contrasts of fat and casein has yet to be used to discriminate between them in scattering, which is therefore agnostic to chemical nature in practice. Studies of physicochemically-separated fat and casein do provide partial validation. The size distribution of casein micelles can be fitted using a log-normal form peaked between 100–200 nm.^18,25,26^ This distribution appears not to overlap with the scattering peak centred at ≈4 µm, which therefore can be ascribed exclusively to fat droplets. However, a number of reports suggest that the smaller population of particles detected by scattering from whole milk may be a mixture of casein and fat,^9,10,27^ with perhaps 1–2% of the total fat volume consisting of droplets with size ≲0.5 µm.^27^

Methods exist to separate fat and casein for individual study, but these are not problem-free. For example, surface proteins on fat droplets reduce their density difference with water, rendering smaller droplets nearly neutrally-buoyant^10,26^ and therefore barely separable by centrifugation. Moreover, resuspending sedimented casein micelles can also be challenging,^28^ and sedimentation can alter the micelles.^29,30^ Isolating casein micelles by chemical modification can disrupt them,^31^ while highlighting them by refractive-index matching fat droplets will likely fail because the latter's index depends on size.^10^

Ideally, therefore, one wants a method that can discriminately size both casein micelles and fat droplets without the need for prior separation. Fluorescence is one possibility. However, conventional optical microscopy resolves only down to ∼0.3 µm and therefore cannot size casein micelles and putative smaller fat droplets. ‘Super-resolution’ microscopy using structured illumination can overcome this limitation,^32^ but has only been applied to milk gels to date^33^ and not to liquid milk.

In this work, we show that cryo focussed ion beam (FIB) scanning electron microscopy (SEM) can discriminate between casein micelle and fat by their different electron density, and that sophisticated analysis of cryo-FIB-SEM tomography images^34,35^ can give a size distribution of each component. While SEM has been used before for sizing milk in combination with other techniques,^36,37^ cryo-FIB-SEM tomography is new in this field. We compare the results with those obtained using differential dynamic microscopy (DDM), which sizes particles by imaging their dynamics. The method has conceptual similarity to dynamic light scattering (DLS), which has long been used for particle sizing in milk.^11,26,38^ DDM is simpler to implement; it can cope with a significant amount of sample turbidity^39^ and is particularly good for revealing multimodality in the size distribution,^40^ but is again, in its simplest form, agnostic to chemical identity. DDM has previously been used to monitor the gelation dynamics of milk.^41^

To date, DDM and cryo FIB SEM have seldom, if ever, been used together for particle sizing. We therefore start by offering a brief introduction to both techniques to facilitate understanding by readers from these distinct user communities. Thereafter, we detail their application to milk. We finish by comparing the results obtained from the two methods, from which we offer some general remarks on sizing milk and other industrial suspensions.

## 2 Background

### 2.1 Differential dynamic microscopy

DDM provides size information *via* dynamics.^42,43^ The raw data are time-lapsed videos of the sample, recording the intensity *I*(*r⃑*,t) in the image plane (*r⃑*). So long as particle dynamics cause fluctuations in the image intensity, the method does not require resolving individual particles, and is indeed usually implemented at low magnification without resolving most or any of the particles in order to maximise the depth of field.^40,42,44^ Analysing intensity fluctuations in the video gives dynamical information on the distribution of the diffusivities of Brownian particles, from which a distribution of their sizes can be deduced.

To do so, we calculate difference images at various delay times, *τ*, *D*(*r⃑*,*τ*) = *I*(*r⃑*,*t* + *τ*) − *I*(*r⃑*,*t*), and take their Fourier transform 
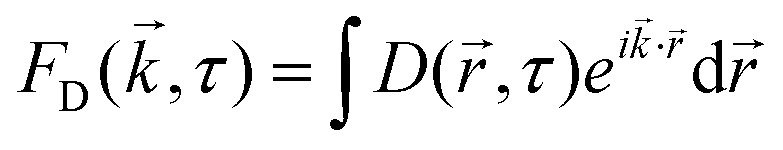
. We then average over the start time *t* and the direction of *k⃑* to obtain the ‘differential image correlation function’ (DICF), *g*(*k*,*τ*) = 〈|*F*_D_(*k⃑*,*τ*)|^2^〉 over a range of *k* values. If the image intensity is proportional to the particle number density, then

1 *g*(*k*,*τ*) = *A*(*k*)[1 − *f*(*k*,*τ*)] + *B*(*k*),

where *A*(*k*) and *B*(*k*) measure signal strength and noise respectively, and *f*(*k*,*τ*) is the so-called intermediate scattering function (ISF)^45^ already familiar from DLS, which contains information about the decay of density fluctuations at length scale 2π/*k*. For a dilute suspension of spheres, we expect^46^2
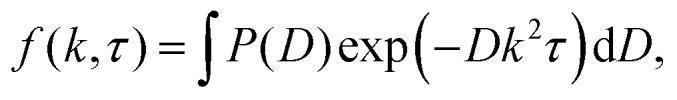
where *P*(*D*) is the probability density function of the single particle diffusivity 
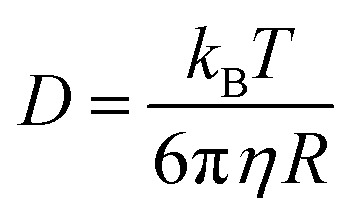
, with *R* the particle radius, *k*_B_*T* the thermal energy and *η* the solvent viscosity.

To obtain *P*(*D*) from the measured ISF, any of the numerical inversion methods familiar from DLS can be used, such as CONTIN.^40,47,48^ However, DDM offers advantages for sizing multimodal suspensions.^40^ In particular, at the *k* values typical of DLS, the single-particle form factor varies significantly with *k*, so that different particle sizes become near-invisible at particular angles. DDM for sizing operates at much lower *k*, where the form factor of all species are constant and their contribution depends only on size.

### 2.2 Cryo-FIB-SEM tomography

**Fig. 1:**
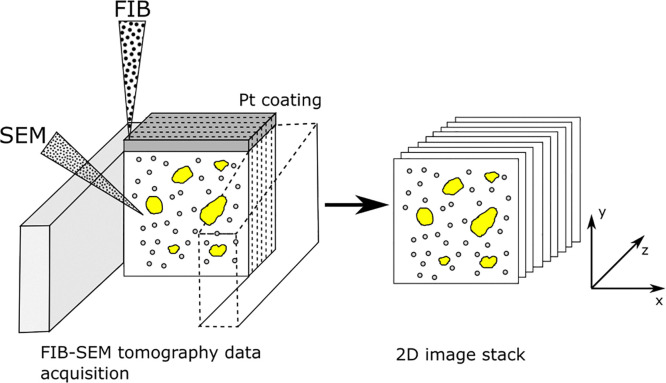
FIB-SEM tomography acquisition (left) is achieved by the FIB removing thin layers of material from a cross section of the sample. After each layer is removed, the SEM takes an image of the cross section, resulting in an 2D image stack (right) that contains *x*, *y* and *z* information of the volume milled. The image stack is then processed and analysed to characterise the 3D structure within the milled volume. Image inspired by.^52^

FIB-SEM tomography is a well established technique for imaging the 3D structure of materials from the nm to the µm scale.^49–51^ Data are acquired by milling a trench into the sample using a FIB and imaging the cross section with an SEM. Milling off successive layers to acquire an image stack allows 3D reconstruction, Fig. 1.

In cryo-FIB-SEM, soft or liquid samples are first flash-frozen before being FIB sectioned and imaged. Such samples are challenging for SEM because they are typically non-conducting and so prone to charging, easily beam damaged and give low contrast and signal-to-noise ratio (SNR) due to the predominance of lighter elements. Nevertheless, the challenges can be overcome, and the method now routinely produces high-quality reconstructions of cells and tissues,^34,35^ although the possibility of ice damage in water-rich samples remains a possibility that needs to be guarded against.

## 3 Materials and methods

### 3.1 Samples

Commercial homogenised milk was locally procured on the day of experiments and refrigerated until use. All samples originated from one of the largest processing plants in Scotland supplied by over 200 farmers, giving a representative average product that likely reduces variation between batches (see SI). DDM and cryo-FIB-SEM measurements were performed simultaneously on samples from the same stock, so that any variation is due solely to the measurement technique and not sample differences.

### 3.2 DDM

Milk is a dense colloidal suspension, with the casein micelles alone contributing more than 10% of the sample volume.^25^ DDM measures collective dynamics, which for such dense suspensions will be strongly affected by particle interactions. In order to use DDM for sizing it is therefore necessary to dilute the commercial milk samples sufficiently to ensure weak enough interactions for the Stokes–Einstein relation to be applicable. Here, samples were diluted 50× in Milli-Q water and loaded into 0.2 × 4 × 50 mm glass capillaries (Vitrocom Inc), sealing the ends with Vaseline to avoid evaporation and drift. We checked that our results were not affected by sample turbidity^39^ (see SI).

We took brightfield videos on a Nikon Ti–E inverted microscope with a high-speed Hamamatsu Orca Flash 4.0 camera at a height of 100 µm from the base of the capillary. To capture a wide range of decay times and low-*k* dynamics,^40^ 256 × 256 pixel recordings of 20 000 frames were obtained at 100 fps with a 10×/0.3 NA objective, a similar NA condenser and 2 × 2 pixel binning, giving an effective pixel size of 1.3 µm. Using the classical depth of field^53^ of ≈10 µm as an estimate for the effective sample height, this corresponds to an imaged volume of ≈10^6^ µm^3^. Measurements were performed in triplicate.

The DICF was extracted using previously-described software.^54^ We selected the range 0.55 µm^−1^ ≤ *k* ≤ 1.5 µm^−1^ for analysis because in this range, enough of the high/low time plateaus of the DICF are present to extract *A*(*k*) and *B*(*k*) reliably.^40^ From this the ISF is calculated and passed to the CONTIN algorithm^47,48^ to be fitted with 60 logarithmically-spaced bins to obtain *P*(*D*) over 0.01 µm^2^ s^−1^ ≤ *D* ≤ 500 µm^2^ s^−1^.

Direct integration was used so that the sum of bin heights (rather than bin areas) describes the contribution, avoiding logarithmically-scaled bin widths hiding populations.^47,48^ The regularisation strength was selected using CONTIN's default method of comparing the impacts of uncertainty and regularisation.^47^ Further details have been given before.^40^

### 3.3 Cryo-FIB-SEM

#### 3.3.1 Sample preparation

Milk was pipetted into 3 mm Type B aluminium carriers and frozen using a Leica EM HPM100 High Pressure Freezer. Cooling at >2 × 10^4^ K s^−1^ minimised freezing damage. Samples were transferred at −196 °C into the cryo-prep chamber, which together with the SEM stages were maintained at −140 °C. The sample, revealed by breaking the specimen carriers, was sublimed at −90 °C for 5 min to remove surface frost and ice, sputter-coated with ≈10 nm platinum, and transferred into the SEM chamber.

#### 3.3.2 Imaging

We used a Zeiss Crossbeam 550 cryo-FIB-SEM with a Quorum Technologies PP3010T cryo system. To reduce vertical striations in the milled cross section (‘curtaining’),^51,55^ an additional 1–2 µm of platinum was deposited onto the frozen sample surface using the Gas Injection System prior to FIB milling, with the precursor reservoir set to 30 °C to minimise sample heating during the ≈40 s deposition process. Initial trenches of depth >10 µm were milled using a 30 kV, 3 nA ion probe, with the current reduced to 300 pA for subsequent milling of 30 nm slices for tomography acquisition. To maximise detected electrons from the bottom of the cross section, the initial mill and trench were wider and deeper than the field of view.^51^

Serial section imaging was carried out using a 2 kV probe at 50 or 100 pA. Rather than seeking to minimise sample charging,^56^ we chose an accelerating voltage to induce a little water charging to make it appear brighter.^34^ This enhances casein micelle contrast, but gives the fat droplets larger charging ‘halos’. We used an InLens SE detector with a drift-compensated frame integration (*N* = 40) to acquire 2048 × 1536 pixel images with 7.5 × 7.5 nm^2^ pixels and 7.5 × 7.5 × 30 nm^3^ voxels. We limited the imaging volume to ≈15 × 11 × 9 μm^3^ for whole milk and ≈15 × 11 × 6 μm^3^ for skimmed milk to achieve the resolution necessary to resolve the casein micelles within a reasonable acquisition time, which constrained the number of fat droplets that could be characterised.

#### 3.3.3 Analysis

To extract particle sizes, image stacks were preprocessed to remove vertical ‘curtaining’ stripes, flat field corrected to remove intensity variations in the background due to local charging effects, and aligned to remove the effect of stage drift.

The fat globules had enough contrast to be easily extracted using a simple Otsu threshold^57^ after image smoothing. Casein micelles have much lower contrast, and must be segmented differently. In skimmed milk, a Phansalkar threshold^58^ was applied. This is an extension of Sauvola's threshold designed to separate foreground and background in low contrast greyscale images. Charging halos around the fat globules in whole milk made segmentating casein micelles more challenging, requiring a combination of both Otsu and Phansalkar thresholding (detailed in the SI). After thresholding, manual corrections were carried out to both data sets to remove obvious artefacts. These were not exhaustive, so that some artefacts undoubtedly remain.

After segmentation, the total volume of each particle was determined by counting the voxels. An equivalent radius for a sphere of the same volume was then calculated.

## 4 Results

### 4.1 DDM

**Fig. 2:**
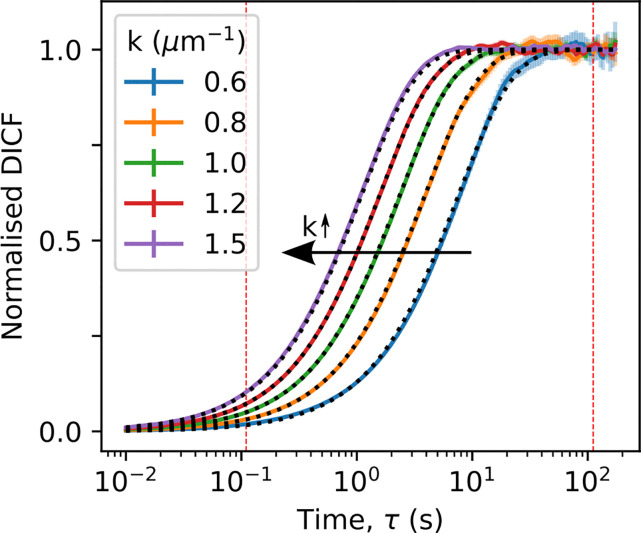
DDM extracted DICF from whole milk videos, normalised as [*g*(*k*,*τ*) − *B*(*k*)]/*A*(*k*), for five Fourier components (*k*) in the selected range. Error bars are standard error from azimuthal averaging. Black dotted lines are CONTIN fits. DICF values to the left and right of the red dotted lines are used to estimate *A*(*k*) and *B*(*k*) respectively as described in.^40^

**Fig. 3:**
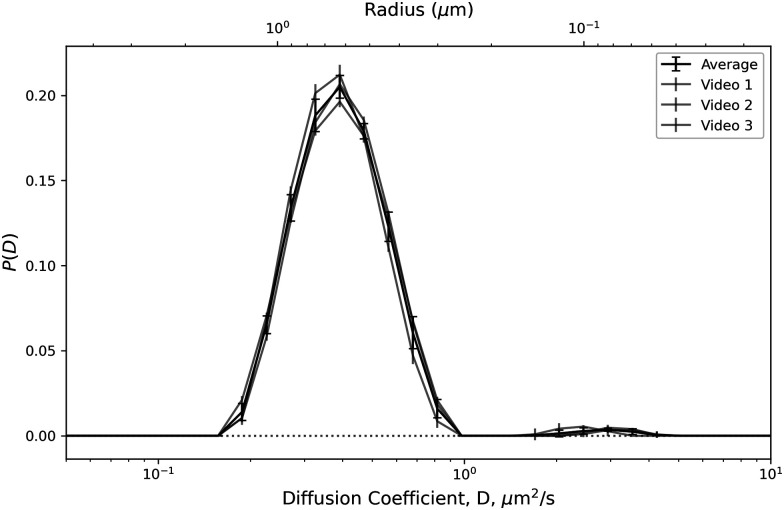
Diffusivity distributions *P*(*D*) extracted by CONTIN for each of the three replicates (light lines) and the average (black line) with error bars indicating CONTIN uncertainty^47,48^ and standard deviation respectively. The range of *D* plotted is set based on accessible range, see SI.

Visual inspection of typical DICFs extracted from our whole-milk data, Fig. 2 (points), shows that they include enough of the short- and long-time plateaus to enable robust fitting to obtain *A*(*k*) and *B*(*k*), eqn (1). From the CONTIN fits of eqn (2), Fig. 2 (lines), we obtain the diffusivity distribution function. Fig. 3 shows that results from three measurements using comparable regularisation parameters are consistent, giving an average that is clearly multimodal.

Peaks occur at *D* = 0.4 µm^2^ s^−1^ and 3 µm^2^ s^−1^, corresponding to (*R*^6^ weighted^40^) radii‡‡From here onwards, all sizes are radii unless otherwise state. of 600 nm and 80 nm. The peaks contribute 98.7% and 1.1% of the signal respectively. The latter contribution is smaller than our previously-noted limit for CONTIN identification of populations.^40^ However, in the present case, the relative size ratio is much larger and the density of the suspension (and the associated signal) much higher than previously, so that, in accordance with our suggestion in the former work, the detection limit should improve. This is confirmed by the fact that the fitted populations do not vary dramatically between repeats. A potentially artificial population at *D* = 25 µm^2^ s^−1^ (contributing 0.2% of the signal) has been omitted from the plot, since diffusion coefficients outside the limits on Fig. 3 are not reliably accessible with this frame rate and *k* range (see SI for details).§§A further reason why the small peak at *D* = 25 µm^2^ s^−1^ may be an artefect is that even a small degree of turbidity can also contribute signal at very short time scales.^39^

The two modes in Fig. 3 correspond well with expectations for fat globules and casein micelles. However, we cannot confidently assign these peaks to these species based on this data because, as already noted, the fat droplet size distribution almost certainly has a ‘tail’ that overlaps with the upper end of the size of casein micelles^9,10,27^ and our analysis cannot distinguish between signal from fat and from protein.

Results obtained for skimmed milk depended on magnification and field of view, likely due to a very small number of large (relative to casein) fat globules still present.^40^ We will not present or discuss these data further (see SI for details).

### 4.2 Cryo-FIB-SEM tomography

**Fig. 4:**
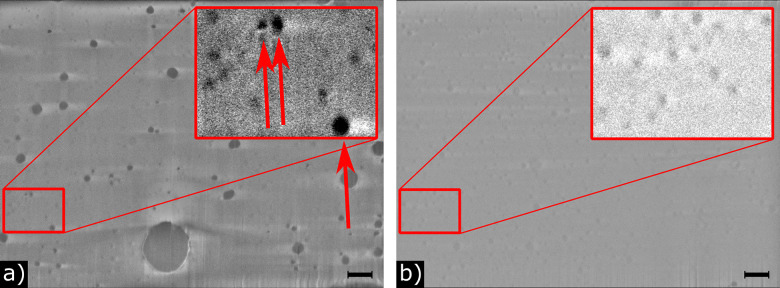
Destriped and flatfield corrected images of (a) whole and (b) skimmed milk, obtained by cryo-FIB-SEM. Scale bars indicate 1 µm. Insets are 3× magnified images with increased contrast. Arrows highlight dark contrast particles, which we take to be fat globules.

Contrast in FIB-SEM cross sections of frozen soft material is poorly understood. However, in cross sections of cells, lipid-rich structures appear dark while the water-rich areas appear bright.^34^ Hence, fat droplets should appear darker than heavily-hydrated micelles.^4,59^ This allows us to visually distinguish fat droplets and casein micelles in our SEM images, Fig. 4. In the image for whole milk, Fig. 4(a), the dark, high-contrast particles with a wide range of sizes are fat droplets, and the much lower contrast particles (only apparent in the enhanced contrast insets) are casein micelles. Importantly, scanning through the sections of any one of these high- or low-contrast particles, we find that its contrast remains unchanged. Thus, the differing contrast in any one image slice is not an artefact of sectioning leaving behind different portions of particles. From the whole milk images it is clear that the casein micelles are on average much smaller than the fat droplets. However, we can also immediately see (Fig. 4(a) [inset]) that there are fat droplets and casein micelles with comparable intermediate sizes. Note that this overlap in size distribution can be qualitatively confirmed by confocal microscopy using two different dyes to label casein and fat, respectively (see SI).

**Fig. 5:**
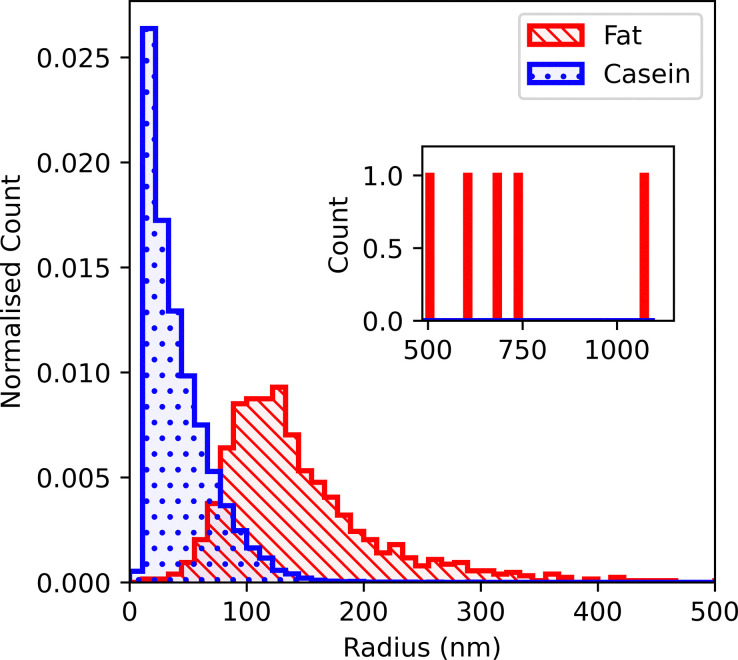
Size distribution of particles with low-contrast (casein micelles) and high-contrast (fat globules) in whole milk, obtained by SEM. Note that each individual distribution is normalised so that the area sums to unity. Inset: Unnormalised distribution above 0.5 µm radius, where only 5 fat globules were observed.

Particle size analysis of our FIB-SEM image stacks (as outlined in section 3.3.3) confirms and quantifies these visual observations. Fig. 5 shows the two size distribution for whole milk, obtained from analysing 1153 fat and 20 640 casein particles, with an inset extending the distribution to beyond 1 µm, where we observe only 5 fat droplets.

Note first that the two distributions heavily overlap in the range 50 nm ≲ *R* ≲ 100 nm. Secondly, the micelle distribution is very much sharper than the fat droplet distribution, and is strongly asymmetric. The large number of very small particles (comparable to a few voxels) is likely noise misidentified as particles, which, despite in-depth image processing, are difficult to distinguish from micelles due to their low contrast.

**Fig. 6:**
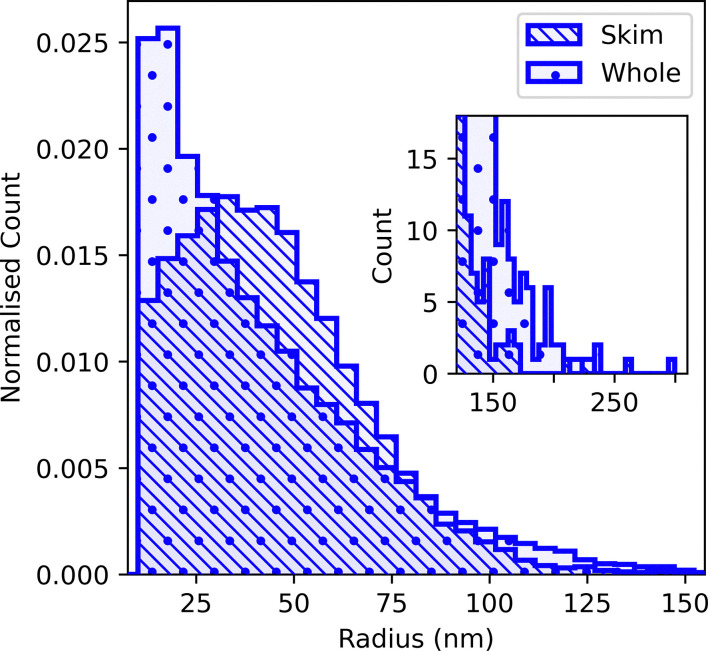
Size distribution of low-contrast particles (casein micelles) in skimmed milk compared to that obtained for whole milk, re-shown here from Fig. 5 for comparison. Note that each individual distribution is normalised so that the area sums to unity. Inset: Unnormalised distribution for high sizes, showing a small number of casein micelles with diameters up to and above 500 nm.

It is possible that at least some of the noise misidentified as very small casein micelles in whole milk originates from charging artefacts associated with fat droplets. To test this hypothesis, we turn to skimmed milk, where the vast majority of particles have similar contrast, Fig. 4b. Three darker fat globules were manually identified and omitted from the analysis. Counting the remaining 11 591 particles gives a casein micelle size distribution for skimmed milk, Fig. 6 in which there is no sharp peak at small sizes.

We also find a number of casein micelles with *R* ≳ 100 nm in whole milk that are not present in skimmed milk, Fig. 6. Possibly, some of these correspond to previously-reported giant spherical micelles (see, *e.g.*, Plate 1a in ref. 60). Interestingly, the sphericity of the largest of the particles identified as micelles with *R* > 200 nm ranges from 0.38 to 0.65, compared to 0.85 ± 0.1 for the whole of this population, which may indicate the presence of non-spherical micellar aggregates.^61^ However, some of the largest particles identified as casein in whole milk may also be artefacts (see SI for details), which is why we consider the sizes extracted from the skimmed milk sample more reliable for further analysis.

### 4.3 Comparing DDM and cryo-FIB-SEM

Different sizing techniques seldom, if ever, yield the same answer. Instead, comparing the results may constrain what could legitimately be inferred from any single technique, and perhaps give further insights not obtainable from using each technique in isolation. To do so, however, requires care to ensure that one is in fact comparing the comparable.

Most importantly, results must be put into a common form for comparison. In our case, SEM gives a number-weighted size distribution, the physical meaning of which is clear, but can be heavily biased by very small artefacts that contribute little volume. On the other hand, for DDM at low *k*, the contribution of a particle scales as

3 *A*(*k*) ∝ (Δ*n*)^2^*R*^6^,

where Δ*n* is the refractive index difference between particle and fluid.^40^ This weighting can directly be compared to DLS results, but is less meaningful than either number- or volume-weighted distributions. We therefore convert both SEM and DDM results into volume-weighted distributions, which are directly useful for understanding suspension properties that depend on volume fraction.

**Fig. 7:**
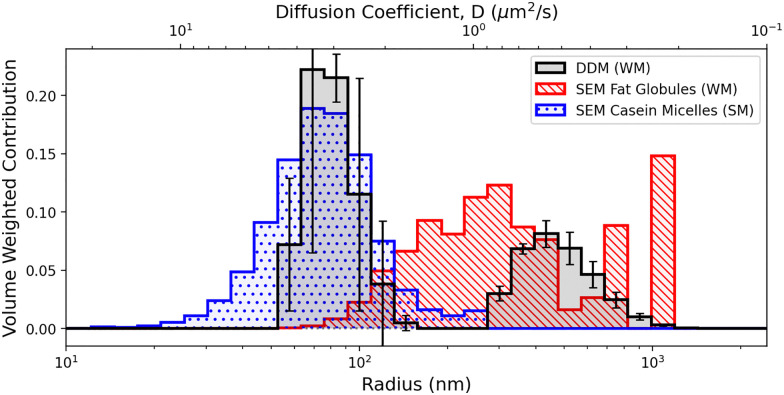
Size distributions for particles in milk as obtained by DDM and cryo-FIB-SEM. Both distributions have been converted to volume weighting, ‘WM’ and ‘SM’ distinguish data from whole and skim milk respectively. Error bars on DDM data show standard deviation between repeats. Note that each individual distribution is normalised so that the bins sum to unity.

Such conversion is straightforward for the SEM data, but doing so for the DDM data requires knowledge of refractive indices; we use 1.46 for fat^62,63^ and 1.49 for casein.¶¶The average of two literature values, *n* = 1.57^64^ and *n* = 1.41.^62^ SEM reveals overlapping casein and fat distributions, so that each of the two populations identified by DDM contains both species. However, multiple uncertainties within each method and in their interrelationship preclude the use of the SEM data to model the refractive indices of the DDM populations. Instead, we assume that the larger population identified by DDM (*D* < 1 µm^2^ s^−1^) is entirely fat and the smaller population (*D* ≥ 1 µm^2^ s^−1^) is entirely casein to convert the *R*^6^-weighted diffusivity distribution, Fig. 3, into a volume-weighted size distribution for comparison with the corresponding distribution obtained from SEM, Fig. 7.

The peak positions at ≈80 nm radius from the two methods agree well. However, the larger population in DDM peaks at ≈400 nm, which is somewhat larger than the peak at ≈300 nm for the SEM fat-globule peak. Moreover, the SEM peak occurs where DDM finds few or no particles.

Comparing the volume-weighted mean radii from DDM and SEM, Table 1, the results for the larger population are well within experimental uncertainties.||||Uncertainty in DDM is the standard error across the three repeats. Repeat is not possible for cryo-FIB-SEM tomography. The error here is estimated as the standard deviation over this data set and two identical data sets where the largest particle has been duplicated or removed. This is significantly larger than a putative standard error of the mean, which, however, is inapplicable because these distributions are non-Gaussian. The DDM result for the average micelle radius (from whole milk) agrees well with the average micelle radius from SEM in the case of skimmed milk. We think there are at least two reasons why comparing to the SEM average micelle radius from whole milk is less appropriate here. First, imaging micelles in whole milk is more challenging than in skimmed milk: the presence of the much darker fat droplets and the associated charging effects make it much harder to image the micelles in whole milk with sufficient contrast for reliable image analysis. Second, our results for whole milk suggest the presence of non-spherical micellar aggregates. We find that if we exclude particles with sphericity <0.65 from the SEM data, the average volume-weighted radius drops to 86 nm.

**Table 1:** Volume weighted mean radii

Population	DDM	cryo-FIB-SEM (nm)
Fat (whole milk)	455 (8) nm	430 (80)
Micelles (whole milk)	80 (7) nm	100 (10)
Micelles (skim milk)	N/A	82 (13)

One initially surprising aspect of Table 1 is that the average micellar size obtained from SEM is larger than that from DDM, even if we take the value of *R* = 86 nm for the more spherical particles from SEM. DDM measures the hydrodynamic radius of particles *via* the diffusivity. For ‘hairy’ particles like casein micelles,^7,30,65^ the hydrodynamic radius likely exceeds the geometric radius,^66^ while the micellar core probably gives the highest contrast in SEM.^66^ Furthermore, diluting milk with water, as we did for DDM measurements but not for SEM sample preparation, will increase its pH towards 7, which previous work suggests will increase the micellar size^67^ (although over a timescale much longer than our experiment). All of these factors lead us to expect a smaller SEM size than DDM.

However, these apparent discrepancies simply highlight the difficulty of characterising suspensions with wide particle size distributions as well as the inherent strengths and limitations of DDM and FIB-SEM. The DDM analysis presented here returns a population averaged diffusivity distribution with a signal weighting inherently biased towards the largest particles in the size distribution (see eqn (3)). Due to the relatively large sample volume probed, the results are statistically robust and DDM can reliably identify a bi-modal size distribution for the whole milk sample. However, as extracting the particle size distribution requires to solve an inverse problem (eqn (2)), it struggles to resolve the detailed shape of the distribution. This is at least in part traceable to the fact that the width of peaks returned by CONTIN is set by the regularisation parameter, which is selected based on uncertainties in the DICF, the estimation of which remains an active area of research.^68–70^ Typically, regularisation leads to a substantial underestimation of the polydispersity by favouring sharper peaks separated by near-zero regions.

Conversely, FIB-SEM provides detailed information on individual particles, including a simple distinction between casein micelles and fat droplets. This can be used to construct number-based size distributions, clearly highlighting an overlap not detected by the DDM analysis. However, statistics is inherently limited, as the high resolution needed to detect casein micelles together with laborious image stack acquisition means that it is only feasible to image relatively small sample volumes. This makes it a poor technique to estimate volume-weighted characteristics, as the conversion increases weight of particles in the large radius tail of the distribution.

## 5 Summary and conclusions

We have characterised the particles in whole and skimmed milk using a combination of DDM and cryo-FIB-SEM. DDM uses standard laboratory equipment and is high throughput. It returns a bimodal distribution, but does not distinguish between casein and fat. cryo-FIB-SEM uses highly-specialised equipment. Even without quantitative analysis, processed images indicate that while the larger population found by DDM consists of fat droplets, the smaller population must be a mixture of casein and fat. Quantitative comparison was performed by converting both DDM and SEM data into volume-weighted size distributions. There is good agreement in the averages, but current methods of data analysis in DDM have a tendency to underestimate polydispersity. While it may be tempting to take DDM results as suggesting that there are no fat globules with radii below 250 nm, we can see from SEM that ≲40% of the volume actually comes from fat particles below this cutoff. Nevertheless, and somewhat remarkably, there is reasonable agreement in the volume-averaged sizes of casein micelles and fat droplets found by the two methods.

Our work suggests that DDM may be a useful method for obtaining a fast characterisation of particle sizes in milk, especially if only averages are important; but detailed interpretation of the results needs to be informed by investigation using imaging methods. In particular, we have demonstrated that cryo-FIB-SEM can provide a detailed characterisation of the particles in milk. Beyond milk, our approach may be applicable to other complex suspensions found in many applications such as food, personal care and pharmaceuticals that are multimodal in size and complex in terms of chemical composition.

## Author contributions

JJB: conceptualization, methodology, software, formal analysis, investigation, writing – original draft, writing – review & editing, visualization. FHJL: methodology, software, validation, formal analysis, investigation, resources, data curation, writing – original draft, writing – review & editing. TP: methodology, validation, formal analysis, investigation, resources, data curation, writing – review & editing. NYDL: methodology, software, validation, formal analysis, investigation, resources, data curation, writing – original draft, writing – review & editing, visualization, project administration. AKB: conceptualization, methodology, formal analysis, investigation, writing – review & editing. TG: methodology, formal analysis, investigation, supervision. VAM: conceptualization, methodology, writing – review & editing, visualization, supervision. JA: methodology, software, writing – review & editing, supervision. JHJT: conceptualization, methodology, validation, writing – review & editing, supervision, project administration, funding acquisition. WCKP: conceptualization, methodology, writing – review & editing, supervision, project administration, funding acquisition.

## Conflicts of interest

There are no conflicts to declare.

## Supplementary Material

SM-022-D5SM01201E-s001

## Data Availability

The data corresponding to this paper are available online through Edinburgh DataShare at: https://doi.org/10.7488/ds/8012. Supplementary information (SI) is available. See DOI: https://doi.org/10.1039/d5sm01201e.
