# Supplementary material for: Particle sizing in milk by combined differential dynamic microscopy and cryo-FIB-SEM tomography
Source: Soft Matter. 2026 May 29;22(23):4074–81. doi: 10.1039/d5sm01201e (PMC13220121; doi:10.1039/d5sm01201e)
Supplement: SM-022-D5SM01201E-s001 [file SM-022-D5SM01201E-s001.pdf]

Supplementary Information (SI) for Soft Matter.
This journal is © The Royal Society of Chemistry 2026

Particle Sizing in Milk by Combined Differential Dynamic Microscopy and Cryo-FIB-SEM Tomography - ESI

Joe J. Bradley*[1], Fraser H. J. Laidlaw[1], Tom Pendry[1], Ngai Ying Denise Li[1], Alexander K. Boggon[1,2], Thomas Glen[1,3], Vincent A. Martinez[1,4], Jochen Arlt[1], Job H. J. Thijssen[1], and Wilson C. K. Poon[†1]

[1]School of Physics & Astronomy, The University of Edinburgh, Peter Guthrie Tait Road, Edinburgh EH9 3FD, United Kingdom
[2]Living Systems Institute, University of Exeter, Exeter EX4 4QD, United Kingdom
[3]Rosalind Franklin Institute, Fermi Ave, Didcot OX11 0QS, United Kingdom
[4]Dyneval Ltd., Roslin Innovation Center, Edinburgh EH25 9RG, United Kingdom

1 Milk sample details

Table 1: Main milk constituents per 100ml according to nutritional labels

Constituent	Whole milk	Skimmed milk
Fat	3.7 g	0.1 g
Protein	3.5 g	3.6 g

Commercial homogenised whole and skimmed milk was procured from local supermarkets (Tesco skimmed milk and Sainsbury's whole milk) on the day of experiments and refrigerated until use. All samples originated from the same processing plant, which is one of the largest in Scotland, supplied by over 200 farmers, giving a representative average product that likely reduces variation between batches. For expected composition according to bottle labels, see table 1.

*joe@joe-bradley.co.uk
†W.Poon@ed.ac.uk

2 Cryo-FIB-SEM datasets

2.1 SEM image analysis

Here we give details of how we segment our SEM images into casein and fat droplets. A schematic of the workflow is given in Figure 1.

Image stacks were first decurtained using wavelet-FFT filtering (Matlab, version R2022a; decomposition level 8 with Daubechies dB15 wavelets, Gaussian damping value = 2.5) [1]. Background variations from localised water charging were then removed using Matlab's flat field correction function with sigma value 70, and the images drift-corrected using centre of mass drift correction in ThermoFisher's Avizo^{TM} software. Since the surface of the sample is typically not perpendicular to the FIB column, micelles and fat droplets appear stretched after reconstruction. This was corrected by shearing the image stack in the vertical direction until the particles looked spherical (by visual inspection). Before segmentation, images were cropped to remove edge artefacts.

For the skimmed milk sample, although casein micelles have significantly lower contrast in SEM than fat droplets, image analysis was simpler due to less artefacts being present from the lower fat content. Here, the image contrast was first enhanced using ImageJ (version 1.54i). Phansalkar thresholding [2], optimised for low-contrast images, was then employed (radius = 30 pixels, default $k = 0.25$, $r = 0.5$, values chosen by visual inspection) to extract the casein micelles and fat droplets, followed by manual correction using ThermoFisher's Avizo^{TM} software on several hundred images containing several thousand identified particles. Micelles should be circular, so non-circular features were removed. We also removed particles near halos and other charging features that visibly affected the local pixel intensity values. This procedure was not exhaustive, so that minor errors may remain. Finally, morphological opening was used to fill any holes in the identified particles.

In the whole milk sample, the presence of large numbers of fat droplets and the surrounding charging halos required an innovative, two-staged approach, Figure 1. We first seek to identify both fat droplets and casein micelles using one algorithm and the fat droplets alone using another. Then, we subtract these and refine the casein micelles.

In detail, we start by smoothing the image stack using a median filter, which blurred the casein micelles into the background. Otsu thresholding was then employed to segment out the fat globules, followed by manual correction. In parallel, we identified both fat droplets and casein micelles using Phansalkar thresholding on the *unsmoothed* image stack with the same parameters as with skimmed milk. The fat droplets emerging from this procedure differ from those emerging from the Otsu thresholding: they are typically larger and included charging halos. We then carried out a morphological reconstruction using the haloed (Phansalkar) data as masks and the non-haloed (Otsu) data as markers to expand the fat droplets in the latter up to the borders of the former. Finally, we subtracted these morphological-reconstructed fat droplets from the Phansalkar thresholded dataset containing both fat droplets and casein micelles

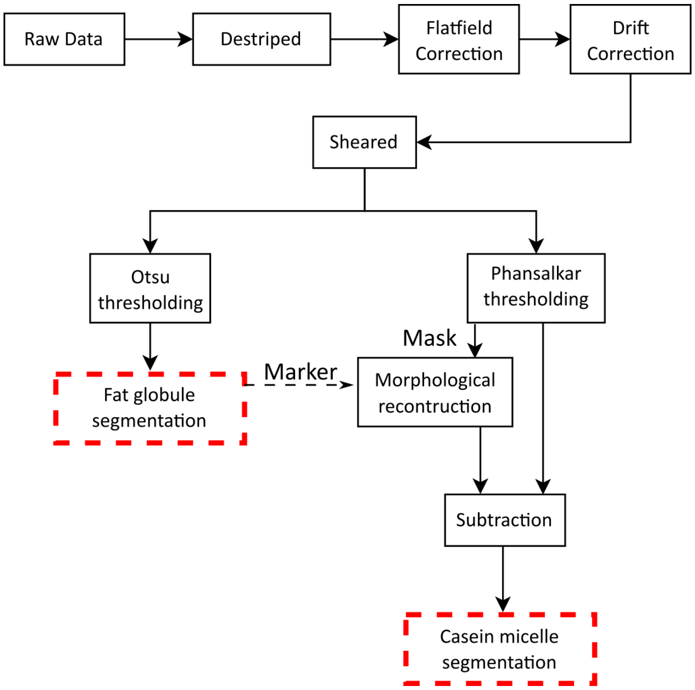

Figure 1: Schematic workflow showing the main steps in analysing whole milk images.

to leave only the latter, which were then manually corrected using the same criteria as for the skimmed milk data.

2.2 Weighted Fat Distributions in Whole Milk

The discrepancies revealed when comparing the size distribution of fat globules obtained from cryo-FIB-SEM and DDM can partly be attributed to the different weightings of the distribution (as discussed in the main text). To demonstrate this, Figure 2 shows the size distribution of fat globules obtained with cryo-FIB-SEM with different applied weightings; that is the raw number weighted distribution, the calculated volume-weighted distribution discussed in the main text, and the calculated intensity, or NR^6, weighted distribution that is relevant to DDM.

Number fluctuations are still clearly visible in the NR^6-weighted distribution, but the contribution of small particles (below a few hundred nm) is almost entirely hidden by the intensity weighting even though they show up clearly in both the volume and number weighted distributions. Moreover, there is a very small but finite contribution in the intensity-weighted distribution of fat globules at $\approx 200\,\text{nm}$ from SEM where the DDM data (Figure 7 in the main text) reports no particles (probably, as discussed in the main text, an effect of regularisation in CONTIN)

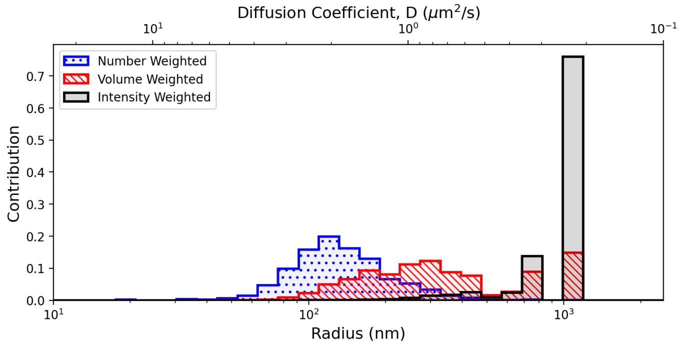

Figure 2: Size distributions for fat globules in milk as obtained by Cryo-FIB-SEM, weighted by number, volume, and intensity. The sum of peak heights describes contribution, each distribution is normalised.

2.3 Artefacts in casein micelle identification

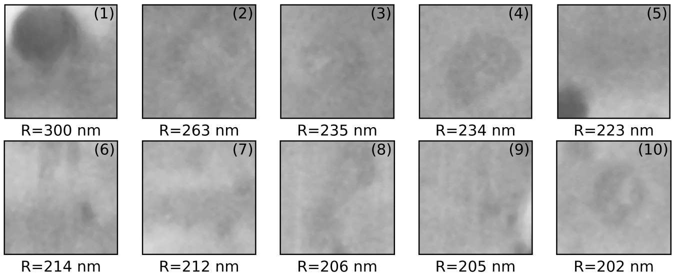

Figure 3: SEM images of the 10 largest particles identified as micelles in analysis of the whole milk sample. Each image has the same contrast as Figure 4 in the main text, and the edges are 840 nm. Frame (1) is clearly a misidentified fat globule. (2) is highly nonspherical and in the 3D stack is more like a sheet or membrane. (3),(4), and (10) are likely micelles. (5)-(8) are artefacts which link visibly to fat globules outside the field of view (e.g. bands visible in Figure 4 in the main text) and (9) is an artefact from milling on that slice.

Identifying casein micelles in the SEM image stack of the whole milk sample was particularly challenging, as the step of removing the fat droplets and their halos can introduce artefacts. We can investigate this by manually inspecting the cryo-FIB-SEM images of the ten largest particles identified as casein micelles in SEM images of whole milk, Figure 3. Of these, the largest is a misidentified fat globule (near the edge of the sample, hence misclassification), three can be confidently described as micelles, five are artefacts, and one is a non-spherical object which looks more like a sheet or membrane – possible residue from globule

membrane damage during homogenisation [3].

2.4 Volume weighted casein micelles size distributions

In the main manuscript we used the casein micelle size distribution obtained from the skimmed milk sample for further comparison with the DDM results.

As we argued there, extracting casein micelles from the whole milk SEM stack is much more susceptible to artefacts. When converting the size distributions to volume-weighting (fig 4), it is the higher number of large micelles in the whole milk sample which distinguish it from the skimmed milk results. Removing some of the artefacts (see previous section) by only including particles with sphericity > 0.65 leads to a very similar distribution to the one we found for the whole milk sample.

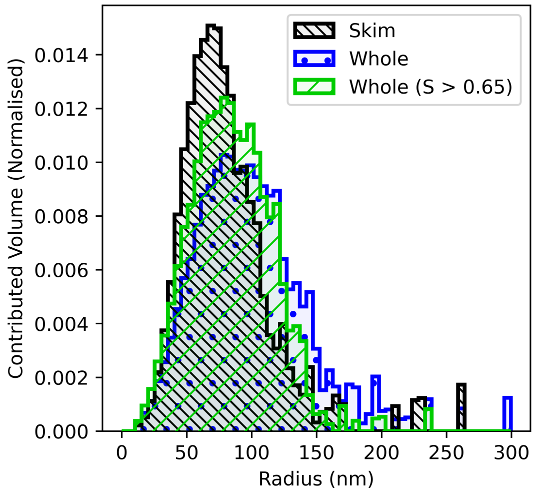

Figure 4: Volume weighted size distribution of casein micelles based on SEM data (as shown in fig. 6 in main manuscript). The whole milk sample appears to have a wider distribution with a larger mean size. However, when considering only particles with sphericity $S > 0.65$ the difference to the skimmed milk distribution is less marked.

3 DDM sample preparation and analysis

3.1 Sample dilution

As commercial milk is a dense colloidal suspension where the casein micelles by themselves already contribute more than 10% to the sample volume, it needs to be diluted in order to be able to deduce particle sizes from the dynamics measured by DDM. We tested various dilutions in our standard sample chambers

(glass capillaries with a height of 200 µm) to identify the dilution where the measured dynamics no longer changed with dilution (here 1:50). Sample turbidity can also introduce artefacts in the measured dynamics, introducing faster decays due to multiple scattering [4]. In order to check that turbidity does not effect our results we verified that using capillaries of only 100 µm height (and therefore reduced turbidity) gave the same dynamics as in our standard geometry.

Here we used milli-Q water to dilute our commercial milk samples. Though it has previously been reported that using water instead of, for example, milk serum [5] can increase the size of the casein micelles [6], we did not observe an increase in size over time in our measurements, presumably because they were all performed within a time window of 20 minutes of dilution, which is substantially shorter than the 24 h equilibration period considered in ref. [6].

3.2 Complete DDM Distributions for whole milk

In the main text, the result is shown over a narrower range than the full CONTIN grid, which is shown in Figure 5. This cropping omits a small population at $D \approx 25\,\mu\text{m}^2\,\text{s}^{-1}$, corresponding to particles with a 10 nm intensity-weighted average radius.

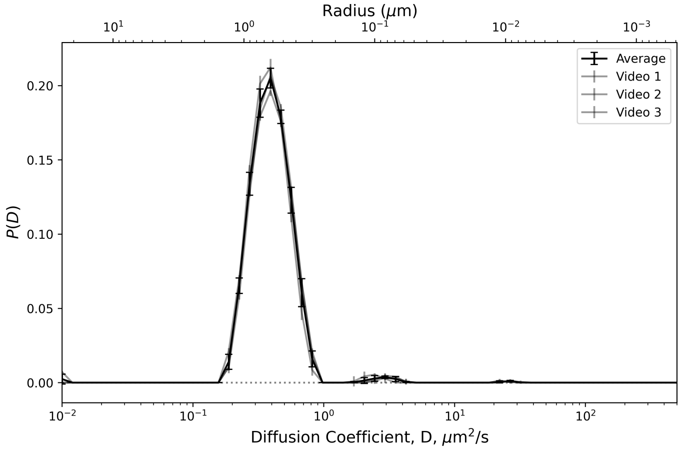

Figure 5: Complete diffusivity distributions $P(D)$ extracted by CONTIN for each of the three replicates (light lines) and the average (black line) with error bars indicating CONTIN uncertainty [7, 8] and standard deviation respectively.

This small peak is consistent between repeats, and there are conceivably particles with comparable sizes within the milk (e.g. whey proteins and micelle subunits). However, the contribution is very small (only 0.2% of the signal), and for this D within our k range, the characteristic timescale for ISF decorrelation is $t_c = (Dq^2)^{-1} \sim 20 - 130\,\text{ms}$. This is detectable at our frame rate of $100\,\text{s}^{-1}$ only for the lowest few k values, assuming a measurement can be made from

only a single datapoint below t_c. It is also possible that even a small degree of turbidity may create short time scale artefacts [4]. We therefore do not discuss this population further.

The limits on Figure 3 in the main text are calculated based on the practicalities of fitting this offset exponential. We suggest that to properly observe the decorrelation of the DICF (a requirement to obtain $B(k)$ for CONTIN), the time between frames must be $\Delta t < t_c/10$ (i.e. less than 10% decorrelation between frames), and the length of the video $t_{max} > 10t_c$. With our average $k = 1\,\mu\text{m}$, $\Delta t = 10\,\text{ms}$, and $t_{max} = 180\,\text{s}$, the reliable range of diffusion coefficients is $0.5 - 10\,\mu\text{m}^2\,\text{s}^{-1}$. Outside of this range, inaccuracies in $B(k)$ estimation will be mistaken for populations with large D (conceivably with t_c on the order of the frame interval, as observed in Figure 5), and inaccuracies in $A(k)$ will be mistaken for populations with very low D (a small flick is also observed in the lowest bin in Figure 5). Turbidity or non-diffusive motion (e.g. any bacteria in the sample) could also introduce false peaks in the CONTIN distribution.

3.3 DDM on skimmed milk

While CONTIN analysis of the whole milk DDM signal gave reliable and reproducible results, we found that this was not the case for the movies we had recorded with the skimmed milk samples. Here the PSD was not consistently bimodal and had a mean size which was sensitive to the field of view. We believe this is because skimming milk does not just remove fat droplets randomly, but is heavily biased towards removing large fat droplets (since they cream faster). This means that the remaining droplets are mostly of the small and intermediate size but still of reasonable number and the distribution likely has a tail. More of this tail is captured as the field of view is increased. In practice it proved difficult to record a large enough field of view to sample the tail of the fat droplet distribution sufficiently while still having high enough spatial resolution to detect the signal from casein micelles. Additionally, the mean separations between casein micelle and fat peak is smaller in skimmed milk, making CONTIN fitting more challenging [9].

4 Confocal microscopy

Unless otherwise stated, all chemicals were sourced from Sigma-Aldrich. Whole milk was obtained from a local store in the UK and kept at a temperature between 1 and 5°C for a maximum of 7 days before being replaced. Following Gallier *et al.* [10], milk was diluted using Phosphate Buffered Saline (PBS) solution, which was made by dissolving 1 PBS tablet in 200 ml of distilled water. Fat globules were fluorescently stained using a 0.02% v/w solution of Nile Red in dimethylsulfoxide (DMSO) [11]. The DMSO facilitates effective staining of hydrophobic substances in acqueous solvents and was used here at sufficiently low concentrations to avoid adverse effects on fat [12]. 0.75 ml of Nile Red in DMSO solution, 0.5 ml of milk and 4.5 ml of PBS were mixed in a

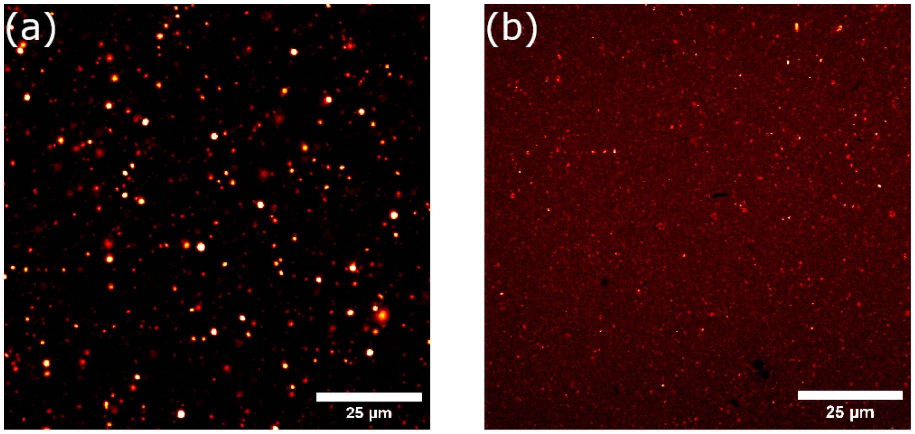

Figure 6: Confocal micrograph of a commercial full-fat homogenised dairy milk fluorescently labelled with (a) Nile Red to show the fat globules, and (b) Rhodamine-B to show the protein (casein) micelles.

glass tube, and placed on a roller mixer (Stuart Roller Mixer SRT9) for 1 hour, resulting in an overall Nile Red concentration of $8.2 \cdot 10^{-5}$ M. In separate but similar samples, protein micelles were stained with Rhodamine B [13]. 2 mg of Rhodamine B was added to 5 ml of milk and 45 ml of PBS, followed by 2 h of roller mixing, resulting in an overall Rhodamine B concentration of $8.4 \cdot 10^{-5}$ M.

A Zeiss LSM700 confocal microscope was used for confocal imaging. Milk samples were gently sandwiched between a microscope slide and a cover slip sealed with double sided sellotape. A 555 nm laser was used to excite both the Nile Red stained samples and the Rhodamine B stained samples. A 63$\times$ air-immersion objective was used for the Nile Red stained samples, while a 63$\times$ oil-immersion objective was used for the Rhodamine B stained ones. Scan times were reduced to minimise blurring due to particle motion.

The images obtained, Fig. 6, confirm that there are two kinds of particles of intermediate size present.

References

[1] B. Münch, P. Trtik, F. Marone, and M. Stampanoni. Stripe and ring artifact removal with combined wavelet - Fourier filtering. *Optics Express*, 17:637, 2009.

[2] N. Phansalkar, S. More, A. Sabale, and M. Joshi. Adaptive Local Thresholding for Detection of Nuclei in Diversity Stained Cytology Images. *IEEE International Conference on Communications and Signal Processing*, pages 218–220, 2011.

[3] M.-C. Michalski, F. Michel, D. Sainmont, and V. Briard. Apparent ζ-potential as a tool to assess mechanical damages to the milk fat globule membrane. *Colloids and Surfaces B: Biointerfaces*, 23(1):23–30, 2002.

[4] R. Nixon-Luke, J. Arlt, W. C. K. Poon, G. Bryant, and V. A. Martinez. Probing the dynamics of turbid colloidal suspensions using differential dynamic microscopy. *Soft Matter*, 18:1858–1867, 2022.

[5] C. G. de Kruif and Thom Huppertz. Casein Micelles: Size Distribution in Milks from Individual Cows. *Journal of Agricultural and Food Chemistry*, 60(18):4649–4655, 2012.

[6] Nidhi Bansal Hotnida Sinaga and Bhesh Bhandari. Effects of milk pH alteration on casein micelle size and gelation properties of milk. *International Journal of Food Properties*, 20(1):179–197, 2017.

[7] S. W. Provencher. A constrained regularization method for inverting data represented by linear algebraic or integral equations. *Computer Physics Communications*, 27(3):213–227, 1982.

[8] S. W. Provencher. CONTIN: A general purpose constrained regularization program for inverting noisy linear algebraic and integral equations. *Computer Physics Communications*, 27(3):229–242, 1982.

[9] Joe J. Bradley, Vincent A. Martinez, Jochen Arlt, John R. Royer, and Wilson C. K. Poon. Sizing multimodal suspensions with differential dynamic microscopy. *Soft Matter*, 19:8179–8192, 2023.

[10] S. Gallier, D. Gragson, R. Jiménez-Flores, and D. Everett. Using Confocal Laser Scanning Microscopy To Probe the Milk Fat Globule Membrane and Associated Proteins. *Journal of Agricultural and Food Chemistry*, 58(7):4250–4257, 2010.

[11] A. G. Barros, J. Liu, G. A. Lemieux, B. C. Mullaney, and K. Ashrafi. Chapter 13 - Analyses of C. elegans Fat Metabolic Pathways. In J. H. Rothman and A. Singson, editors, *Caenorhabditis elegans: Cell Biology and Physiology*, volume 107 of *Methods in Cell Biology*, pages 383–407. Academic Press, 2012.

[12] P. V. Dludla, B. Jack, A. Viraragavan, C. Pheiffer, R. Johnson, J. Louw, and C. J. F. Muller. A dose-dependent effect of dimethyl sulfoxide on lipid content, cell viability and oxidative stress in 3T3-L1 adipocytes. *Toxicology Reports*, 5:1014–1020, 2018.

[13] L. Ong, R. R. Dagastine, S. E. Kentish, and S. L. Gras. Microstructure of milk gel and cheese curd observed using cryo scanning electron microscopy and confocal microscopy. *LWT - Food Science and Technology*, 44(5):1291–1302, 2011.
